# Formulation-Level Tolerability of Repeated Drinking-Water Administration of a Commercial Multi-Component Bacteriophage Product in Ross 308 Broiler Chickens: A Pilot 27-Day Dose-Escalation Study

**DOI:** 10.3390/vetsci13080730

**Published:** 2026-07-24

**Authors:** Ádám Kerek, Zsolt Abonyi-Tóth, Péter Ferenc Dobra, Henrik Baranyay, Tamás Maklári, Ákos Jerzsele

**Affiliations:** 1 Department of Pharmacology and Toxicology, University of Veterinary Medicine Budapest, H-1078 Budapest, Hungary; jerzsele.akos@univet.hu; 2 National Laboratory of Infectious Animal Diseases, Antimicrobial Resistance, Veterinary Public Health and Food Chain Safety, University of Veterinary Medicine Budapest, H-1078 Budapest, Hungary; 3 Department of Biomathematics and Informatics, University of Veterinary Medicine Budapest, H-1078 Budapest, Hungary; toth-abonyi.zsolt@univet.hu; 4 Department of Pathology, University of Veterinary Medicine Budapest, H-1078 Budapest, Hungary; dobra.peter@univet.hu; 5 EQ-Flow Kft., H-2094 Nagykovácsi, Hungary; baranyay.henrik@gmail.com; 6 Micromir Hungary Kft., H-1121 Budapest, Hungary; tmaklari@gmail.com

**Keywords:** bacteriophage, Fagovet, broiler chicken, target-animal safety, dose escalation, drinking water, commercial bacteriophage formulation, poultry, antibiotic alternatives, feed intake

## Abstract

Background: Bacteriophage-based interventions are increasingly considered antibiotic-sparing tools in poultry production, but target-animal tolerability should be documented before efficacy-oriented field application. Methods: This pilot study evaluated the formulation-level tolerability of the repeated drinking-water administration of Fagovet, a commercial multi-component bacteriophage product, in Ross 308 broiler chickens under non-challenged conditions. According to the manufacturer’s product documentation, the formulation contains a declared mixture of 23 bacteriophages at a concentration of not less than 1 × 10^6^ plaque-forming units per milliliter. However, the concentration of active bacteriophages was not independently confirmed immediately before administration or after dilution in drinking water. A total of 120 birds were allocated to 12 pens, with three replicate pens per treatment, and received untreated water or product-volume-based administration rates corresponding to 1×, 3×, or 5× the manufacturer-recommended rate for 27 days. Body-weight gain and pen-level feed intake were analyzed using linear mixed-effects models with Dunnett-adjusted comparisons against controls. Clinical observations, mortality, diagnostic necropsies, terminal gross postmortem examination of all surviving birds, and descriptive histopathology in eight randomly selected birds, comprising two birds per treatment group, were used as supportive endpoints. Results: Body-weight gain did not differ from controls at 1× (ratio 0.99; 95% CI 0.88–1.10; *p* = 0.9856), 3× (1.03; 0.92–1.15; *p* = 0.8273), or 5× (1.01; 0.91–1.13; *p* = 0.9902). Feed intake showed no significant treatment-related reduction, and no consistent dose-related adverse clinical or pathological pattern was identified. Conclusions: Repeated drinking-water administration of the tested commercial formulation was well tolerated under the conditions of this pilot study. Because individual phage components, genomic safety, actual PFU-based exposure, and drinking-water stability were not independently characterized, the findings apply to the administered formulation and should not be interpreted as a component-level safety or quantitative phage dose–response assessment.

## Simple Summary

Bacteriophages are viruses that infect bacteria and are being developed as antibiotic-sparing tools for animal production. This pilot study evaluated the formulation-level tolerability of the repeated drinking-water administration of Fagovet, a commercial multi-component bacteriophage product, in Ross 308 broiler chickens. A total of 120 birds were assigned to untreated control pens or to pens receiving the product at the manufacturer-recommended administration rate, or at three- or five-fold multiples of that rate, for 27 days. Body weight, feed intake, clinical health, mortality, gross postmortem findings, and supportive histopathological observations were assessed. No treatment-related reduction in body-weight gain or feed intake and no consistent dose-related adverse clinical or pathological pattern were detected. The administered dose levels were based on product volume rather than on an independently confirmed bacteriophage concentration in the drinking water. In addition, the individual bacteriophage components were not separately characterized by genomic analysis or isolate-level host-range testing. The findings should therefore be interpreted specifically as preliminary, short-term tolerability data for the tested commercial formulation under non-challenged broiler conditions. They support the design of subsequent studies incorporating quantitative phage exposure, drinking-water stability, microbiological efficacy, and pathogen-challenge endpoints.

## 1. Introduction

Food-producing animals are a central interface between antimicrobial use, bacterial ecology, food safety, and public health. The increasing global burden of antimicrobial resistance has reinforced the need for intervention strategies that reduce reliance on conventional antimicrobial treatments without compromising animal health and welfare [1,2,3,4,5]. In poultry production, this need is particularly clear because enteric and systemic bacterial diseases remain economically important, while poultry meat and eggs are also major vehicles for foodborne zoonoses [6,7,8,9,10]. Recent Hungarian and Central European veterinary studies further indicate that food-animal bacterial populations, including poultry-associated *Escherichia coli* and chicken-origin opportunistic bacteria, may harbor diverse antimicrobial-resistance and virulence-associated traits, strengthening the rationale for preventive, non-antibiotic approaches compatible with food-animal production systems [11,12,13,14,15].

Non-typhoidal *Salmonella* remains among the most important foodborne pathogens associated with poultry and poultry-derived foods. Although vaccination, biosecurity, hygiene, and integrated farm-to-slaughter control programs have substantially improved *Salmonella* control in many settings, the persistence of *Salmonella* in production environments and the occurrence of antimicrobial-resistant strains continue to drive interest in complementary non-antibiotic tools [9,16]. This interest is part of a broader movement in veterinary medicine toward nutritional, biological, and microbiome-compatible alternatives that may reduce antimicrobial dependence without impairing production traits [17]. Among such tools, bacteriophages offer a biologically targeted approach because they infect bacteria through host-specific interactions and can be selected for activity against defined bacterial targets [18,19,20,21,22].

Experimental and applied studies have shown that bacteriophage preparations can reduce *Salmonella* contamination or colonization in poultry-related matrices, including live-bird and food-chain contexts [23,24,25,26,27,28]. However, the effectiveness of phage-based interventions depends on multiple factors, including the target bacterial strain, phage–host range, timing and route of administration, environmental stability, bacterial susceptibility, and the composition of the phage cocktail [29,30]. Therefore, bacteriophage-based approaches should be evaluated in a stepwise manner: in vitro characterization and host-range screening should be followed by target-animal safety evaluation, and then by efficacy studies under challenge or field conditions.

Target-animal safety is particularly important for preparations intended for repeated oral administration in young broilers. Although bacteriophages are generally regarded as highly host-specific and several studies have reported favorable safety profiles in animals and humans [31,32,33,34,35,36], each formulation and route of administration requires independent evaluation. In broilers, safety assessment should include growth performance, feed intake, clinical observations, mortality patterns, and postmortem examination because adverse effects may appear as reduced weight gain, altered feed consumption, poor flock uniformity, or non-specific clinical abnormalities [37,38,39,40,41].

The present pilot study evaluated the formulation-level tolerability of repeated drinking-water administration of the identified commercial multi-component bacteriophage product Fagovet in Ross 308 broiler chickens. The central aim was not to test therapeutic or microbiological efficacy, nor to establish the genomic safety or component-specific properties of individual bacteriophages, but to determine whether daily exposure to the commercial formulation at the manufacturer-recommended product-volume rate and at three- and five-fold multiples of that rate adversely affected body-weight gain, feed intake, clinical health, mortality, or postmortem findings under non-challenged rearing conditions. Exploratory pre-study in vitro assays were summarized solely as product-level activity checks and not as validated potency, host-range, or quantitative titer measurements.

## 2. Materials and Methods

### 2.1. Study Design and Ethical Approval

The study was designed as a controlled, pen-replicated, pilot dose-escalation target-animal tolerability trial in broiler chickens. The animal experiment was conducted at the animal facility of the Department of Pharmacology and Toxicology, University of Veterinary Medicine Budapest, Hungary, under animal experimental authorization PE/EA/150-7/2021. The study was classified as mild because the test formulation was administered orally via drinking water and the birds were subjected only to routine handling, body-weight measurement, clinical observation, and standard husbandry procedures. The study is reported in accordance with the ARRIVE 2.0 guidelines [42].

A total of 123 Ross 308 broiler chickens of mixed sex were obtained from a commercial hatchery. Three chicks died during the pre-allocation period because of early chick weakness and were not included in the experimental cohort. The remaining 120 birds were allocated to 12 pens with 10 birds per pen. The 12 pens were assigned to four treatment groups, with three replicate pens per group: untreated control, 1× dose, 3× dose, and 5× dose. The study period lasted 27 days. The birds had received standard hatchery vaccinations before arrival. Feed and water were offered *ad libitum* throughout the experiment.

### 2.2. Commercial Bacteriophage Formulation and Dosing

The test item was Fagovet, a commercial multi-component bacteriophage formulation intended for oral administration in poultry via drinking water. The product used in the study was declared by the Hungarian distributor to be identical to the complex bacteriophage-containing veterinary product with therapeutic effects marketed under the trade name Fagovet, for which the first marketing authorization was granted in 2019. The manufacturer was OOO NPC MikroMir, Russia, 107031 Moscow, 5/23 Nizhniy Kiszelnij Lane, Building 1, and the Hungarian distributor was Micromir Hungary Kft., 1121 Budapest, Csorna utca 5., 4th floor, Apt. 11, Hungary. The product is registered in Hungary under the following NÉBIH ÁTI registration numbers: 1282/1/2019 NÉBIH ÁTI (2 mL), 1282/2/2019 NÉBIH ÁTI (5 mL), 1282/3/2019 NÉBIH ÁTI (10 mL), 1282/4/2019 NÉBIH ÁTI (50 mL), 1282/5/2019 NÉBIH ÁTI (100 mL), and 1282/6/2019 NÉBIH ÁTI (1000 mL).

According to the manufacturer’s declaration and product specification, Fagovet is a sterile physiological suspension containing bacteriophages, with sterile physiological saline solution as the carrier medium. The bacterial names listed in the product information indicate the target bacterial spectrum of the formulation and do not indicate the presence of viable bacteria in the product. The formulation is described as a sterile, non-toxic, clear light-yellow liquid, with a pH of 7.2–7.4. The manufacturer reports an Appelman bacteriophage activity of 10^−9^ under its product-specific test conditions. This manufacturer-declared value was not independently reproduced or quantitatively verified in the present study. The formulation is stored at +4 °C to +10 °C. The batch used in the present study was Batch No. 200920, manufactured on 20 September 2021, with an expiry date of 20 September 2022.

At the time of the animal experiment, the product was supplied for controlled research evaluation and was tested as the administered commercial formulation rather than as a collection of individually characterized bacteriophage isolates. Individual phage identifiers, isolate-level host-range profiles, electron-microscopy morphology, genomic sequences, and genome-based assessments of lysogeny, transduction potential, or undesirable genes were not available for the present study. The manufacturer-declared number and concentration of bacteriophages were available from product-specific documentation, but the active bacteriophage concentration was not independently confirmed immediately before administration or after dilution in drinking water. Stability and persistence of infective bacteriophages in the daily drinking-water preparations were also not measured. Accordingly, the test item was identified and evaluated at the commercial formulation level, and the study should not be interpreted as a safety evaluation of defined individual phages or as a quantitative PFU-based exposure study. The available manufacturer-declared product specifications are summarized in Table 1.

The manufacturer-recommended administration rate was 1 mL of Fagovet per 100 L of drinking water. Birds in the treated groups received the formulation daily in freshly prepared drinking water at product-volume-based rates corresponding to 1×, 3×, or 5× this recommended rate. Because a validated quantitative bacteriophage titer was not obtained immediately before administration or after dilution in drinking water, these dose levels represent multiples of the administered product volume and not independently verified PFU/mL or PFU/bird exposure levels. The dosing solution was first prepared as a stock dilution in 100 mL of drinking water and was then mixed into the full daily drinking-water volume. Dosing volumes were adjusted according to the age-related water allocation used during the study (Table 2). Individual water consumption was not measured; exposure therefore occurred at pen level and may have varied among birds.

Drinking-water administration was selected because it represents the intended and practically relevant route for flock-level application of the commercial product in poultry. Individual crop gavage could have provided more uniform administered volumes per bird, but repeated gavage would have introduced additional handling stress and an artificial exposure procedure that does not reflect routine broiler production use. The present design therefore prioritized tolerability assessment under the intended route of administration, using replicate pens for each treatment group. The control group received untreated drinking water.

### 2.3. Housing, Feeding, and Environmental Monitoring

Birds were housed in pens designed to meet animal-welfare requirements for the study. The room temperature, relative humidity, and photoperiod were set according to broiler husbandry recommendations. Temperature was gradually reduced during the early phase of the experiment; later in the study, room temperature stabilized at approximately 25–27 °C, because the increasing heat production of the growing birds limited further reduction without excessive reduction in relative humidity. Temperature and relative humidity were recorded in each pen on scheduled measurement days.

Birds were fed a coccidiostat-free, crumbled intensive broiler starter diet. According to the feed specification, the diet contained 10.64% moisture, 20.96% crude protein, 6.00% crude fat, 2.86% crude fiber, 6.07% crude ash, 12.03 MJ/kg apparent metabolizable energy, 1.25% lysine, 0.50% methionine, 1.05% calcium, 0.75% phosphorus, and 0.16% sodium. Fresh feed was provided daily. Feed allocation increased with age: 1.0 kg per pen during the first four days, 1.5 kg per pen during days 6–13, and 2.5 kg per pen during days 15–27. Feed remaining on scheduled measurement days was weighed, and pen-level feed consumption was calculated by subtraction. The complete environmental monitoring dataset is provided in Table S1.

### 2.4. Exploratory Pre-Study Product-Level In Vitro Activity Checks

Exploratory pre-study in vitro assays were performed as functional product-level activity checks before in vivo administration. These assays were not designed or validated as formal product-release potency tests, did not characterize individual bacteriophage components separately, and did not assess stability after dilution in drinking water. Broth-based inhibition testing was performed using a two-fold dilution series of ten vials of the formulation against a *Salmonella* culture. Lytic activity was explored using an Appelmans-type serial dilution assay. Quantitative bacteriophage concentration was attempted using a Gratia double-layer agar approach, and product-level lytic activity against the bacterial lawn was screened by spot testing. Because the Appelmans-type and plaque assays did not provide reliable quantitative results and spot testing is inherently qualitative, these experiments were interpreted only as exploratory evidence of formulation-associated activity. They were not used to establish the administered PFU concentration, individual phage–host range, product potency, or in vivo efficacy. The assays are summarized in Table 3.

### 2.5. Body-Weight Measurement, Feed-Intake Assessment, Clinical Observations, and Pathological Examination

Birds were individually identified within pens by leg marking and weighed on study days 0, 1, 4, 6, 8, 11, 13, 15, 18, 20, 22, 25, and 27. Thus, repeated body-weight measurements could be linked to the same bird over time within each pen.

Feed consumption was measured at pen level on the same scheduled measurement days. Feed intake per bird was calculated by dividing pen-level feed consumption by the number of live birds in the pen at the time of measurement. All birds were observed clinically throughout the study. Any abnormal clinical signs, morbidity, or mortality were recorded. Birds that died during the experiment were submitted for diagnostic necropsy, and postmortem findings were recorded by a veterinary pathologist.

At study termination, all surviving birds (*n* = 116; control, *n* = 29; 1×, *n* = 29; 3×, *n* = 30; and 5×, *n* = 28) underwent gross postmortem examination. Histopathological evaluation was performed in a subset of eight birds, comprising two randomly selected surviving birds from each treatment group (control, *n* = 2; 1×, *n* = 2; 3×, *n* = 2; and 5×, *n* = 2). Birds were selected at random from the surviving animals within each treatment group. The same tissue panel, comprising the liver, kidney, pancreas, duodenum, jejunum, ileum, and cecum, was collected and examined systematically in all eight birds. Samples were fixed in buffered formalin, embedded in paraffin, sectioned, and stained with hematoxylin and eosin. The veterinary pathologist was not formally blinded to treatment allocation. No predefined numerical lesion-scoring system was applied; lesions were recorded descriptively using routine diagnostic morphological criteria. Histopathology was therefore treated as a supportive descriptive safety assessment rather than as a blinded quantitative toxicopathological endpoint. Raw longitudinal body-weight records are provided in Table S2, and raw pen-level feed-consumption data are provided in Table S3.

### 2.6. Statistical Analysis

The primary safety endpoints were body-weight gain and feed intake. Body-weight gain was calculated relative to day 0. Day 0 measurements served as baseline values and were not included as outcome observations in the log-transformed body-weight-gain model. Because the variance of body-weight gain increased with age, body-weight gain was logarithmically transformed before modeling. A linear mixed-effects model was fitted with treatment as the fixed factor of interest and study day as a covariate. Because birds were individually identified within pens by leg marking, body-weight data were analyzed as longitudinal repeated measurements. Bird identity nested within pen was included as a random effect to account for repeated measurements of the same individual, and pen was included to account for clustering within the pen-replicated design. Treated groups were compared with the untreated control group using Dunnett-adjusted post hoc contrasts. Because the model was fitted on the log scale, treatment effects for body-weight gain are reported as ratios of geometric means relative to controls, with 95% confidence intervals.

Feed intake was available at the pen level. Therefore, the pen was treated as the experimental unit for feed-intake analysis. A linear mixed-effects model was fitted to feed intake per bird with treatment as the fixed factor of interest, study day as a covariate, and pen as a random effect. Dunnett-adjusted post hoc comparisons were used to compare each treated group with the untreated control group. Feed-intake effects are reported as absolute differences from control in g/bird/day, calculated from pen-level feed disappearance and adjusted for the number of live birds, with 95% confidence intervals. The original analysis was performed in R version 4.0.5 using mixed-model and multiple-comparison procedures [43,44,45,46,47]. A *p*-value below 0.05 was considered statistically significant. Because this was a safety and tolerability study rather than a formal equivalence or non-inferiority trial, absence of statistically significant differences was interpreted together with effect sizes, confidence intervals, clinical observations, and postmortem findings. Diagnostic plots used to assess model assumptions are provided in Figure S1.

## 3. Results

### 3.1. Exploratory Pre-Study Product-Level Activity Checks

**Figure 1:**
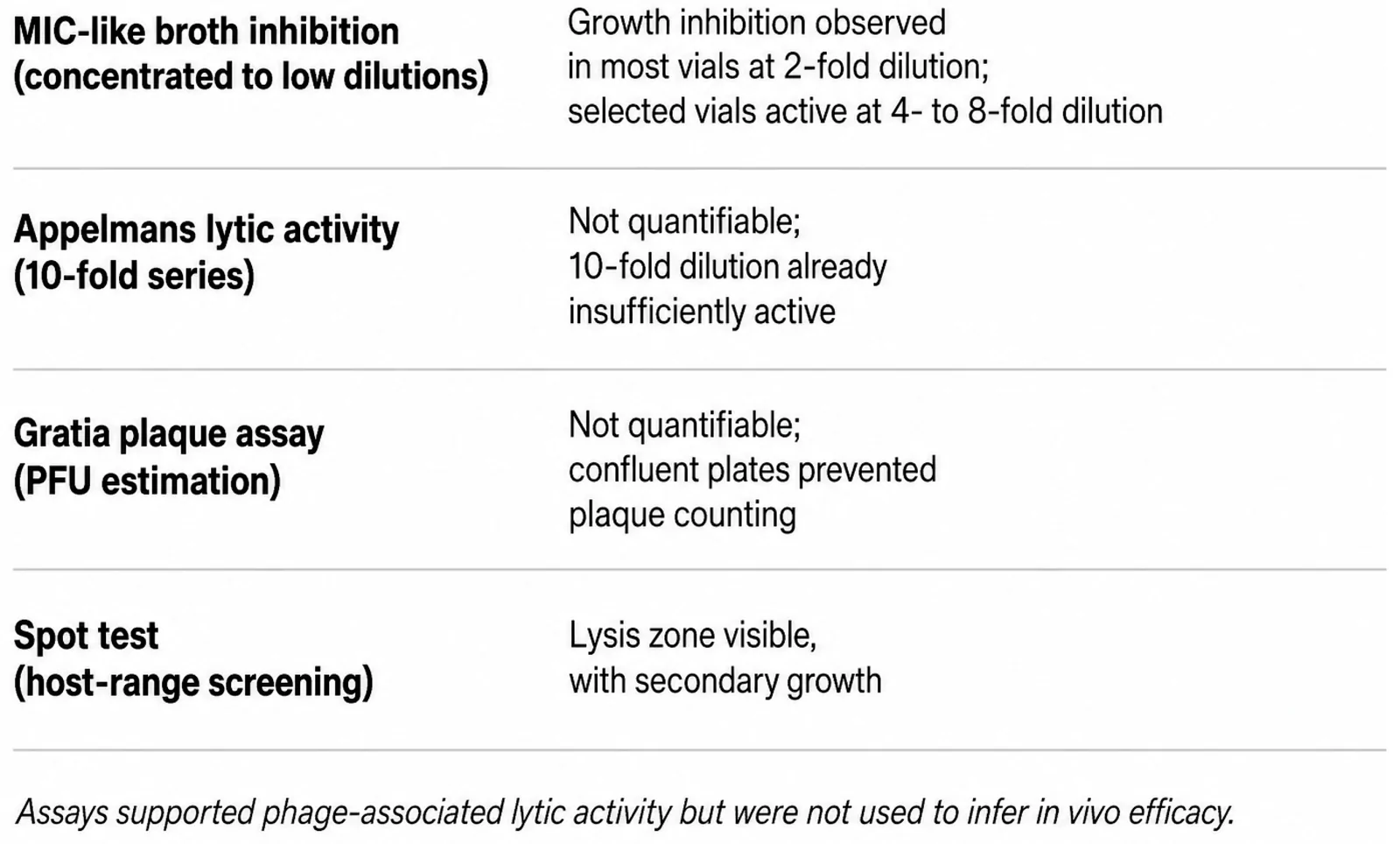
Exploratory pre-study product-level in vitro activity checks of the Fagovet bacteriophage formulation. The assays provided qualitative evidence of formulation-associated activity but did not establish a quantitative bacteriophage titer, component-specific host range, product potency, or in vivo efficacy. MIC: minimum inhibitory concentration.

Broth-based testing indicated phage-associated inhibitory activity against *Salmonella* culture at concentrated or low-dilution levels in most tested vials. In samples 1 and 9, inhibition was observed up to four-fold dilution, and in sample 8, up to eight-fold dilution. The Appelmans-type assay did not allow a reliable determination of lytic activity because activity was not retained at the first-tested ten-fold dilution (Figure 1). The Gratia plaque assay did not yield a quantifiable concentration because plates were confluent. Spot testing showed a visible lysis zone, although secondary growth was present. These findings provided only qualitative evidence of formulation-associated bacteriophage activity. They did not yield an independently validated bacteriophage titer, did not establish the potency of the daily drinking-water preparations, and were not used to infer therapeutic efficacy or component-specific host range.

### 3.2. Environmental Conditions and Study Conduct

**Figure 2:**
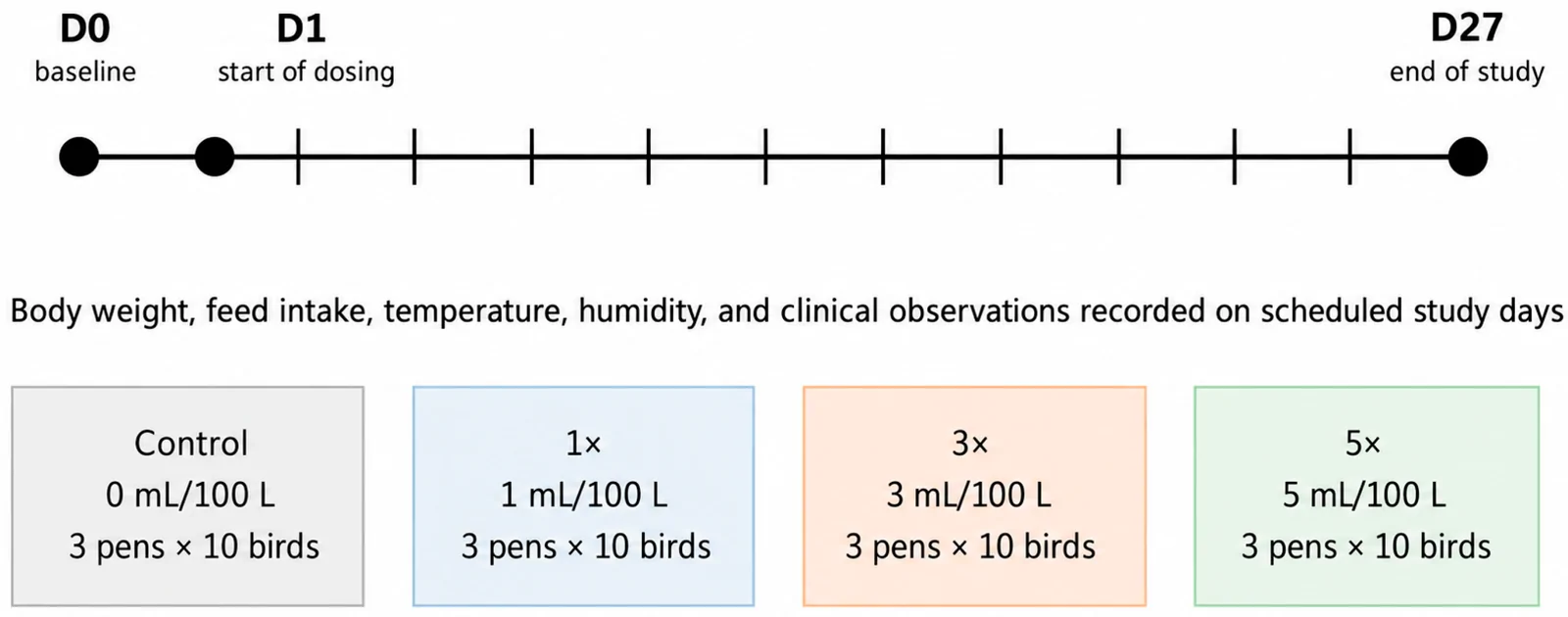
Experimental design and dosing schedule. A total of 120 Ross 308 broilers were allocated to four treatment groups, with three replicate pens per group. The Fagovet formulation was administered daily in drinking water from day 1 through day 27.

The study was completed over 27 days. Temperature was reduced during the early rearing period and later stabilized at approximately 25–27 °C, reflecting the balance between ventilation and preservation of relative humidity. Relative humidity was maintained within the targeted range during the early part of the experiment and declined moderately when ventilation was increased later in the study (Figure 2). No environmental deviation was judged to confound the comparison between treatment groups because all groups were kept under the same room-level husbandry conditions.

### 3.3. Body-Weight Development

**Figure 3:**
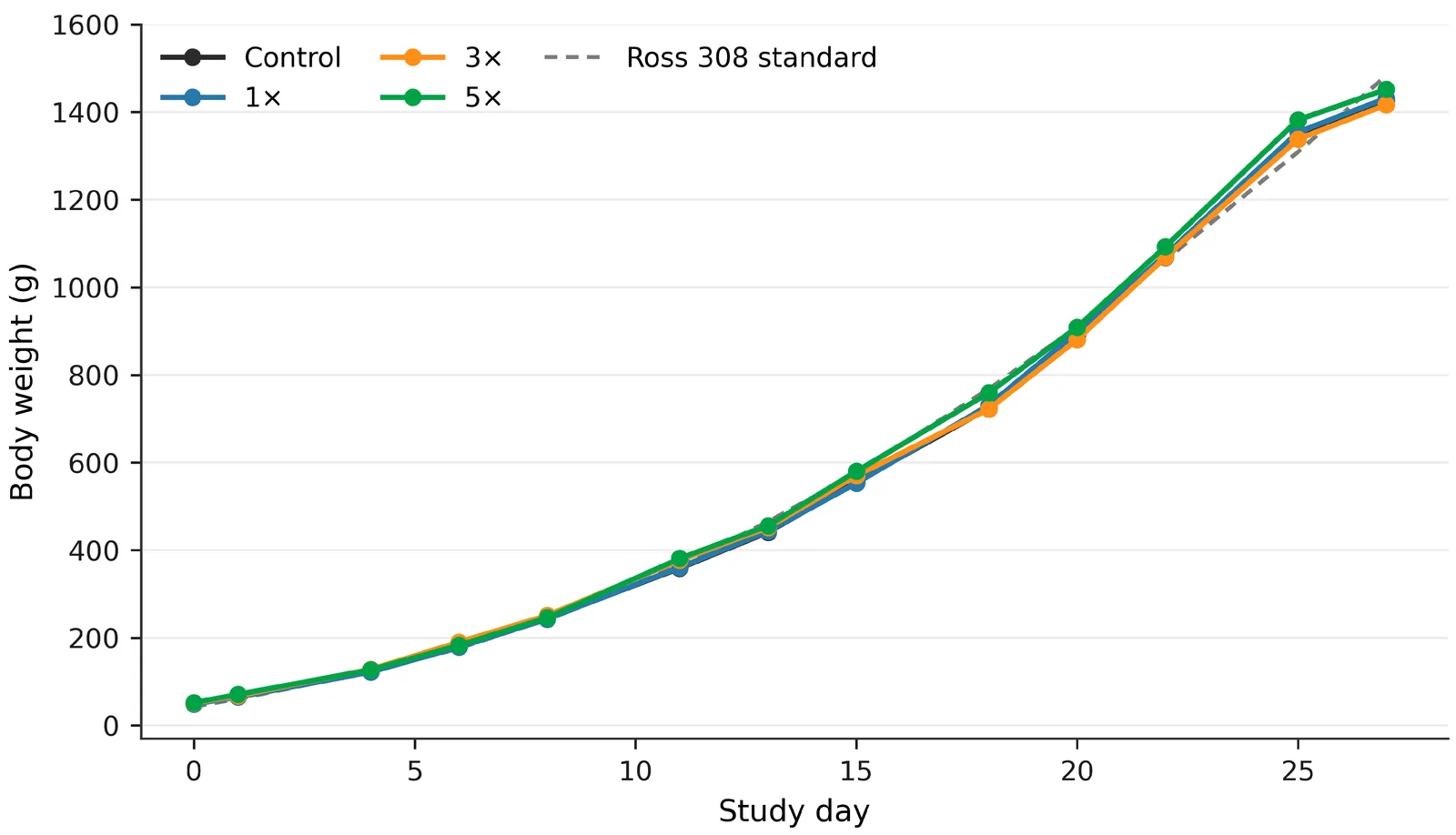
Body-weight trajectories of broiler chickens during repeated drinking-water administration of the Fagovet bacteriophage formulation. Values represent pen-level treatment means with standard errors; the dashed line indicates the Ross 308 performance objective for body weight.

Body weight increased steadily in all treatment groups throughout the study. Treatment-group trajectories were closely aligned with each other, and no dose-dependent depression of growth was apparent (Figure 3). At day 27, treatment-level means calculated from pen-level summaries were similar across groups: 1425.3 g in controls, 1431.7 g in the 1× group, 1416.6 g in the 3× group, and 1451.9 g in the 5× group.

The mixed-effects model confirmed that none of the treated groups differed significantly from the control group in body-weight gain. Relative to controls, the geometric mean ratio for body-weight gain was 0.99 in the 1× group (95% CI 0.88–1.10; *p* = 0.9856), 1.03 in the 3× group (95% CI 0.92–1.15; *p* = 0.8273), and 1.01 in the 5× group (95% CI 0.91–1.13; *p* = 0.9902) (Table 4).

### 3.4. Feed Intake

**Figure 4:**
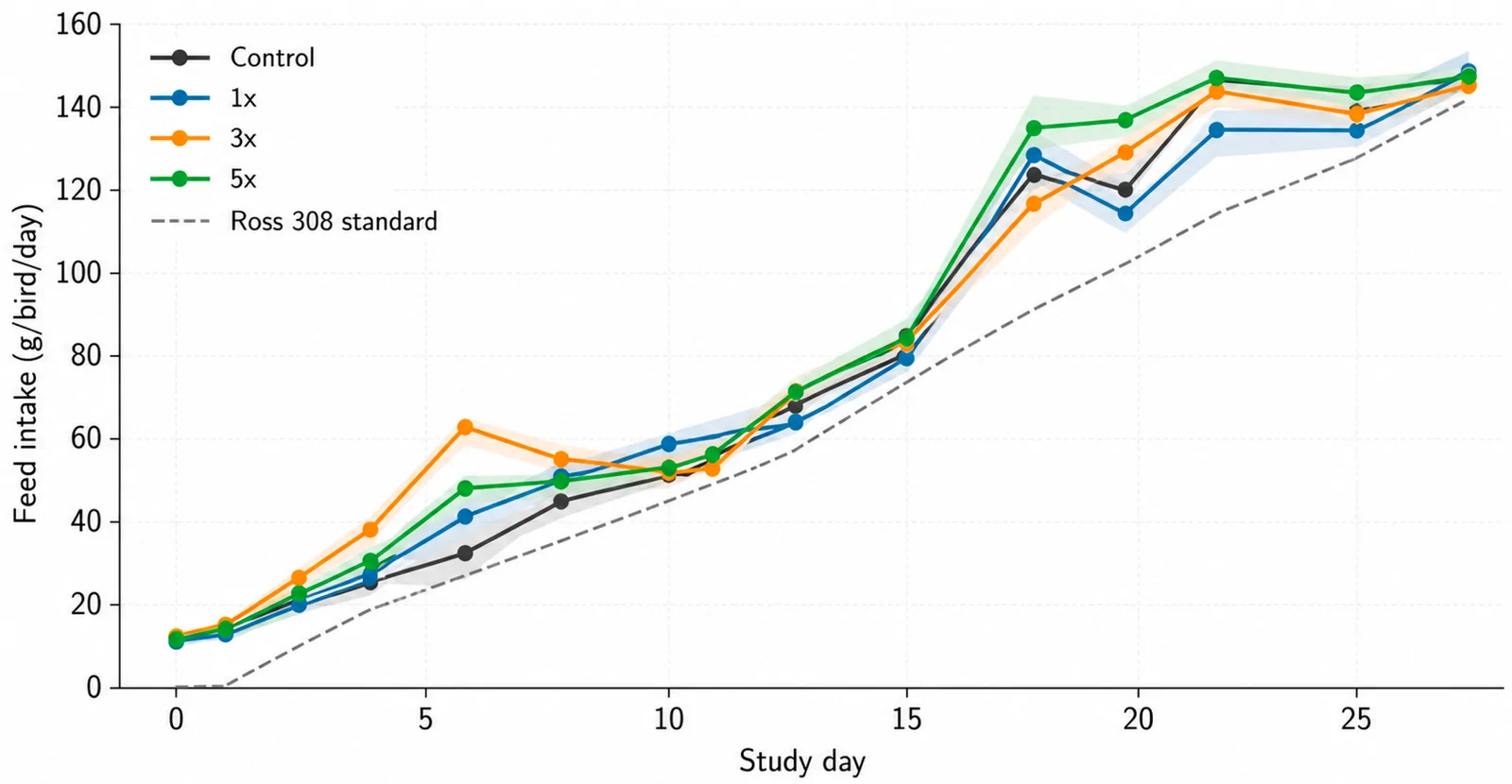
Daily feed intake per bird during the 27-day safety trial. Values represent treatment means based on pen-level observations with standard errors; the dashed line indicates the Ross 308 performance objective for daily feed intake.

Feed intake per bird increased with age in all groups and followed similar temporal patterns across treatments (Figure 4). No dose-dependent reduction in feed intake was observed. At day 27, mean feed intake per bird was 146.9 g in controls, 149.3 g in the 1× group, 147.1 g in the 3× group, and 146.5 g in the 5× group.

**Figure 5:**
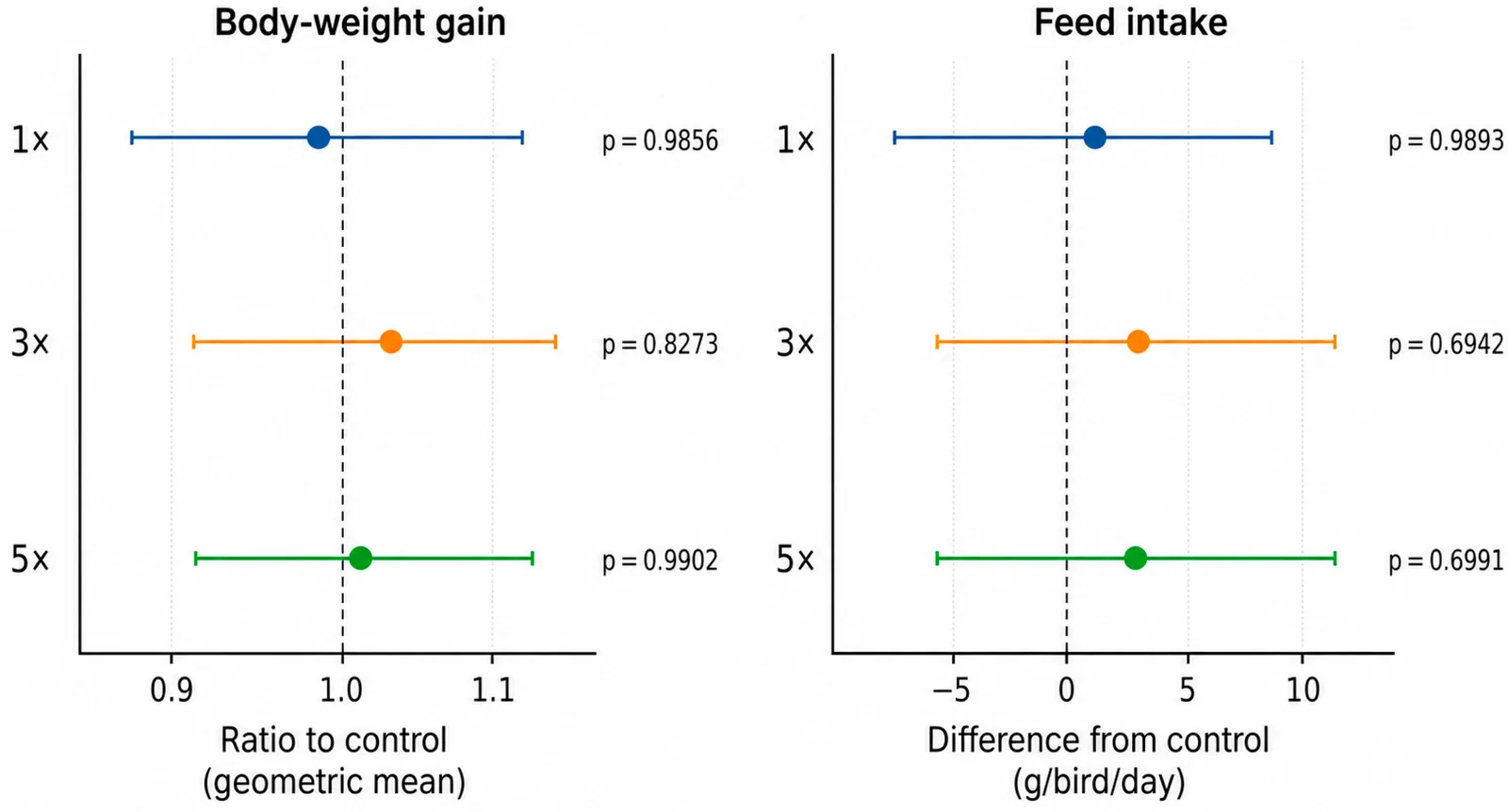
Dunnett-adjusted effect estimates for treated groups compared with controls. Body-weight gain estimates are ratios of geometric means. Feed-intake estimates are absolute differences from control in g/bird/day, calculated from pen-level feed disappearance and adjusted for the number of live birds. Error bars indicate 95% confidence intervals.

The mixed-effects model showed no statistically significant treatment-related change in feed intake. Compared with controls, the estimated difference was 0.78 g/bird/day in the 1× group (95% CI −6.54 to 8.09; *p* = 0.9893), 2.81 g/bird/day in the 3× group (95% CI −4.51 to 10.12; *p* = 0.6942), and 2.78 g/bird/day in the 5× group (95% CI −4.53 to 10.10; *p* = 0.6991) (Table 4 and Figure 5).

### 3.5. Clinical Observations, Mortality, Gross Pathology, and Histopathology

Three additional chicks died before treatment-group allocation because of early chick weakness and were not included in the 120-bird experimental cohort. Splayed legs were occasionally observed, mainly in the later phase of the study, and were considered compatible with non-specific locomotor problems in fast-growing broilers rather than a treatment-related adverse effect. One bird in the 1× group showed depression and impaired movement on day 15. Pecking behavior was noted in one 5× pen on day 27. No consistent treatment-associated clinical syndrome was observed. The complete clinical observation and mortality record is provided in Table S4.

During the 27-day treatment period, four birds died: one in the 1× group on day 5, one in the control group on day 11, and two in the 5× group on days 12 and 13. Diagnostic necropsy identified bacterial septicemic or developmental conditions, including *Escherichia coli* septicemia, *Staphylococcus aureus* septicemia, yolk sac retention with dehydration, and *Streptococcus alactolyticus* septicemia with polyserositis and pneumonia. These findings did not indicate a dose-dependent or formulation-attributable adverse pattern (Table 5). Given the low number of events, mortality was interpreted descriptively rather than subjected to inferential statistical testing.

At study termination, gross postmortem examination was completed for all 116 surviving birds, while descriptive histopathological evaluation was performed in eight randomly selected birds (control, *n* = 2; 1×, *n* = 2; 3×, *n* = 2; and 5×, *n* = 2); no treatment-related or dose-dependent pathological pattern was identified within the histologically evaluated subset. Mild background findings were observed across treatment groups, including variable yolk sac retention, mild pododermatitis, occasional subcutaneous, intramuscular, hepatic, or subepicardial hemorrhages, mild hydropericardium, focal microvesicular hepatic lipidosis, prominent intestinal lymphoid follicles, and mild focal mononuclear infiltrates in parenchymal organs; representative histopathological findings are shown in Figures S2–S5. A single bird in the 1× group exhibited gross inflammatory lesions suggestive of septicemia and was considered an incidental finding; a representative gross image is provided in Figure S6. Intestinal samples were not consistently suitable for detailed villus morphometry because residual intestinal content and autolysis limited reliable evaluation in several sections. Overall, the gross and descriptive histopathological findings did not indicate a consistent formulation-attributable adverse pathological pattern.

## 4. Discussion

This pilot study provides formulation-level target-animal tolerability data for repeated drinking-water administration of Fagovet, an identified commercial multi-component bacteriophage product, in Ross 308 broiler chickens. Daily administration at the manufacturer-recommended product-volume rate and at three- and five-fold multiples of that rate did not impair body-weight gain or feed intake and was not associated with a consistent adverse clinical or pathological pattern. These findings support the short-term tolerability of the administered commercial formulation under the specific non-challenged conditions tested, but they do not constitute a component-level safety assessment of individually defined bacteriophages.

The study was deliberately framed as a safety and tolerability experiment rather than an efficacy trial. This distinction is critical. Bacteriophage efficacy against *Salmonella* or other bacterial targets depends on phage–host matching, route and timing of administration, multiplicity of infection, bacterial susceptibility, gastrointestinal stability, and the ecological context of the gut [19,21,26,29,30]. The present data therefore do not demonstrate pathogen reduction or therapeutic benefit. Instead, they address the preceding translational question: whether repeated oral exposure to the formulation produces measurable adverse effects in the target animal.

The regional relevance of this safety work is strengthened by recent Hungarian and Central European veterinary data showing that food-animal bacterial populations may carry diverse antimicrobial-resistance determinants. Genomic analysis of Hungarian layer-derived *E. coli* isolates demonstrated the value of resistome-level surveillance in poultry, while recent studies on chicken-origin *Proteus mirabilis* and multi-host *Pasteurella multocida* further illustrate that antimicrobial-resistance concerns extend beyond a single bacterial species or production sector [12,15,48]. These observations do not directly predict the efficacy of bacteriophage-based control, but they reinforce the need for antibiotic-sparing interventions whose safety is documented before challenge or field application.

The lack of growth depression is biologically relevant because reduced body-weight gain is among the most sensitive practical indicators of poor tolerability in broiler safety studies. The ratios of geometric mean body-weight gain remained close to unity across all dose levels, and the confidence intervals did not suggest a large adverse effect. Similarly, feed intake did not decrease in any treated group. In broiler production, even modest changes in feed intake can precede or accompany adverse effects, palatability problems, or gastrointestinal intolerance. The absence of a negative feed-intake signal is therefore an important supportive safety observation.

The clinical, gross pathological, and histopathological findings must be interpreted conservatively but are consistent with the performance data. Mortality occurred in both treated and untreated groups and was attributed to sporadic bacterial septicemic or developmental conditions. The occurrence of two deaths in the 5× group required transparent reporting, but the diagnostic necropsy findings did not reveal a common toxicological pattern, and the overall performance of the 5× group was not depressed. Terminal gross postmortem examination of all surviving birds and descriptive histopathological evaluation of eight randomly selected birds, comprising two birds per treatment group, revealed only mild background findings, without a treatment-related or dose-dependent distribution within the evaluated material. One bird in the 1× group showed inflammatory lesions suggestive of septicemia, which was interpreted as an incidental finding rather than a group-level treatment-associated signal. Together, these findings argue against a clinically meaningful dose-related adverse effect of the formulation under the conditions tested. Nevertheless, the pathological evidence should be regarded as supportive rather than definitive because the histopathological assessment was descriptive, no predefined numerical lesion-scoring system was used, and formal blinding was not implemented.

The exploratory pre-study assays provided qualitative evidence of formulation-associated bacteriophage activity but did not yield a validated quantitative titer or a component-level potency assessment. The administered test item was identified as Fagovet, a commercial multi-component bacteriophage product manufactured by OOO NPC MikroMir and distributed in Hungary by Micromir Hungary Kft. Product-specific documentation identified the batch, declared number of component bacteriophages, declared minimum concentration, bacterial target spectrum, carrier medium, pH, sterility, storage conditions, and manufacturer-specified Appelman activity. These data establish the identity and manufacturer-declared specification of the commercial product used in the trial but do not substitute for independent characterization of its individual bacteriophage components.

Individual phage identifiers, isolate-specific host-range profiles, electron-microscopy morphology, genomic sequences, and genome-based assessments of lysogeny, transduction potential, antimicrobial-resistance genes, virulence genes, or other undesirable genetic elements were not available. Consequently, the study cannot establish the genomic safety or strictly lytic nature of each component phage and should not be interpreted as doing so. In addition, no reliable independent titer was obtained immediately before administration, and the infective bacteriophage concentration and stability of the daily drinking-water preparations were not measured. The 1×, 3×, and 5× levels therefore represent product-volume-based multiples of the manufacturer-recommended use rate rather than measured PFU-based exposure levels. The study addresses whether repeated administration of the commercial formulation at these practical use levels produced detectable adverse effects; it does not establish a quantitative bacteriophage dose–response relationship.

Drinking-water administration was deliberately selected to reflect the intended flock-level route of product use in poultry. This approach inevitably allows variation in individual consumption and therefore in individual exposure. Crop gavage could have provided more uniform dosing, but repeated gavage would have introduced handling-related stress and an artificial administration procedure that is not representative of routine broiler production. Accordingly, the present design prioritizes practical route-specific tolerability over precise individual PFU delivery. Future studies should combine drinking-water administration with measurement of individual- or pen-level water consumption, serial quantification of infective bacteriophages in the prepared water, and recovery of bacteriophage activity from gastrointestinal samples.

The broader relevance of these findings lies in the development pathway for phage-based antibiotic alternatives in poultry. Recent work has shown that bacteriophages can reduce Salmonella contamination or colonization in specific settings, and regulatory evaluations have considered bacteriophage feed additives for avian species [19,20,21,23,26]. However, the field still needs well-reported target-animal safety studies that separate tolerability from efficacy claims. By using repeated administration and supra-intended dose levels, the present study contributes safety information for an identified commercial bacteriophage formulation and can support the design of subsequent challenge trials.

Several limitations should be acknowledged. First, this was a pilot, non-challenged tolerability study; microbiological efficacy against Salmonella or any other target organism cannot be inferred. Second, the individual bacteriophage components were not characterized by genome sequencing, component-specific host-range testing, or electron microscopy, and the available data do not allow exclusion of lysogenic, transducing, or genetically undesirable component phages. Third, the manufacturer-declared bacteriophage concentration was not independently confirmed immediately before administration, and bacteriophage stability in the prepared drinking water was not measured. Accordingly, the administered levels represent product-volume-based multiples rather than quantified PFU-based exposures. Fourth, drinking-water administration reflects practical flock-level use but introduces variability in individual exposure because individual water consumption was not measured. Fifth, histopathology was used as a descriptive supportive endpoint; the veterinary pathologist was not formally blinded to treatment allocation, and no predefined numerical lesion-scoring system was applied. Sixth, the trial did not include hematology, serum biochemistry, microbiome sequencing, resistome analysis, immune readouts, or consistently reliable intestinal villus morphometry. Seventh, feed intake was measured at pen level, and the study was not designed as a formal equivalence or non-inferiority trial with pre-specified margins. These limitations restrict the findings to short-term formulation-level tolerability under the specific conditions tested and preclude conclusions regarding individual phage safety, precise phage exposure, product efficacy, or broader field performance.

Future studies should build on these safety results by incorporating a controlled Salmonella challenge, quantification of cecal and environmental bacterial loads, recovery of bacteriophage activity from drinking water and intestinal contents, microbiome and resistome endpoints, and, where accessible, genomic characterization of the component phages. Such studies would allow a direct assessment of whether the formulation can reduce pathogen burden while preserving animal performance and microbiome stability.

## 5. Conclusions

Within the limitations of this pilot formulation-level study, repeated administration of the commercial Fagovet multi-component bacteriophage product via drinking water was well tolerated by Ross 308 broiler chickens over 27 days under non-challenged conditions. Product-volume-based administration at the manufacturer-recommended rate and at three- and five-fold multiples did not significantly affect body-weight gain or feed intake, and the clinical, mortality, gross postmortem, and supportive descriptive histopathological findings did not indicate a consistent treatment-related or dose-dependent adverse pattern. These findings provide preliminary target-animal tolerability data for the administered commercial formulation. They do not establish the genomic safety of individual component phages, independently verified PFU-based exposure, drinking-water stability, or microbiological efficacy. Subsequent studies should incorporate component-level genomic and host-range characterization, validated quantitative titration, stability measurements in drinking water, pathogen challenge, and microbiological and microbiome endpoints.

## Figures and Tables

**Table 1:** Product-level identity and manufacturer-declared specifications of the commercial multi-component bacteriophage product used in the study.

Characteristic	Information
Product identity and type	Fagovet; commercial multi-component bacteriophage-containing veterinary product
Manufacturer/Hungarian distributor	OOO NPC MikroMir, Russia/Micromir Hungary Kft., Hungary
Hungarian registration	1282/1/2019–1282/6/2019 NÉBIH ÁTI
Batch and dates	Batch No. 200920; manufactured 20 September 2021; expiry 20 September 2022
Intended route and administration rate	Drinking water; 1 mL product/100 L drinking water
Active substance	Sterile purified filtrate/suspension of bacterial phagolysates containing bacteriophages
Declared number of bacteriophages	23 component bacteriophages
Declared bacteriophage concentration	Not less than 1 × 10^6^ PFU/mL
Declared bacterial targets	*Escherichia coli*; *Salmonella enterica* spp.; *Pseudomonas aeruginosa*; *Staphylococcus aureus*; *S. epidermidis*; *S. haemolyticus*; *Streptococcus pyogenes*; *Enterococcus faecalis*; *E. faecium*; *E. hirae*; *E. durans*; and *Proteus vulgaris*
Carrier/viable bacterial content	Sterile physiological saline; no live bacteria
Physical and quality specifications	Clear light-yellow sterile liquid; pH 7.2–7.4; non-toxic according to the manufacturer
Manufacturer-specified Appelman activity	10^−9^
Storage conditions	+4 °C to +10 °C
Basis of product control	Manufacturer’s product-specific specification

PFU, plaque-forming units. The number of component bacteriophages, bacteriophage concentration, bacterial target spectrum, Appelman activity, and other product characteristics shown in this table are manufacturer-declared specifications. They were not independently verified at the component level in the present study. The Hungarian registration numbers correspond to the registered package sizes of 2, 5, 10, 50, 100, and 1000 mL.

**Table 2:** Treatment allocation and drinking-water dosing scheme for the Fagovet bacteriophage formulation.

Group	Nominal Dose in Drinking Water	Replicate Pens	Initial Birds	Preparation Scheme
Control	0	3	30	Untreated drinking water
1× dose	1 mL/100 L	3	30	50 µL/5 L on days 1–8
				100 µL/10 L on days 9–15
				150 µL/15 L on days 16–27
3× dose	3 mL/100 L	3	30	150 µL/5 L on days 1–8
				300 µL/10 L on days 9–15
				450 µL/15 L on days 16–27
5× dose	5 mL/100 L	3	30	250 µL/5 L on days 1–8
				500 µL/10 L on days 9–15
				750 µL/15 L on days 16–27

**Table 3:** Summary of exploratory pre-study product-level in vitro activity checks of the Fagovet bacteriophage formulation.

Assay	Approach	Main Outcome	Interpretive Use
Broth inhibition assay	Two-fold dilution series across ten vials.	Most vials inhibited *Salmonella* growth at concentrated or two-fold dilution; selected vials remained active at four- to eight-fold dilution.	Exploratory product-level activity check only.
Appelmans-type assay	Decimal dilution series with culture, phage, and broth controls.	Lytic activity could not be quantified because the first tested dilution already showed insufficient activity.	Qualitative outcome; not used as a titer estimate.
Gratia plaque assay	Double-layer agar plaque assay.	Plaque concentration could not be determined because plates were confluent.	Quantitative titer not available from this assay.
Spot test	Direct application of the formulation to a bacterial lawn.	A lysis zone was visible, with secondary growth.	Screening result only; not interpreted as in vivo efficacy.

**Table 4:** Dunnett-adjusted model estimates for body-weight gain and feed intake. CI: confidence interval.

Endpoint	Comparison	Estimate	95% CI	Adjusted *p*-Value	Scale
Body-weight gain	1× vs. control	0.99	0.88–1.10	0.9856	Ratio of geometric means
Body-weight gain	3× vs. control	1.03	0.92–1.15	0.8273	Ratio of geometric means
Body-weight gain	5× vs. control	1.01	0.91–1.13	0.9902	Ratio of geometric means
Feed intake	1× vs. control	0.78	−6.54 to 8.09	0.9893	Difference, g/bird/day
Feed intake	3× vs. control	2.81	−4.51 to 10.12	0.6942	Difference, g/bird/day
Feed intake	5× vs. control	2.78	−4.53 to 10.10	0.6991	Difference, g/bird/day

**Table 5:** Summary of pre-allocation deaths and treatment-period mortality with diagnostic necropsy findings.

Timing	Group	Number	Main Finding	Interpretation
Pre-allocation	Not assigned	3 chicks	Early chick weakness.	Not included in the 120-bird experimental cohort.
Day 5	1×	1 bird	Yolk sac infection with omphalitis, *Escherichia coli* septicemia, conjunctivitis, and serofibrinous polyserositis.	Sporadic bacterial condition.
Day 11	Control	1 bird	*Staphylococcus aureus* septicemia with yolk sac retention, empty crop, and autolysis.	Occurred in untreated control.
Day 12	5×	1 bird	Splayed legs, yolk sac retention, dehydration.	Non-specific condition; no bacterial septicemia confirmed.
Day 13	5×	1 bird	Serofibrinous polyserositis, hepatomegaly, multifocal bacterial pneumonia, cardiomegaly, *Streptococcus alactolyticus* septicemia.	Sporadic bacterial condition.

## Data Availability

The original contributions presented in this study are included in the article/Supplementary Materials. Environmental monitoring data are provided as Table S1, raw longitudinal body-weight data as Table S2, pen-level feed-consumption data as Table S3, and clinical observation and mortality records as Table S4. Diagnostic plots supporting the model-assumption assessment are provided as Figure S1. Representative histopathological images are provided as Figures S2–S5, and a representative gross pathological image from an incidental individual case is provided as Figure S6. Further inquiries can be directed to the corresponding author.

## Supplementary Materials

The following supporting information can be downloaded at: https://www.mdpi.com/article/10.3390/vetsci13080730/s1, Table S1: Environmental monitoring data; Table S2: Raw longitudinal body-weight data by pen, leg-marked bird number, and study day; Table S3: Raw pen-level feed-consumption data; Table S4: Clinical observation and mortality records; Figure S1: Diagnostic plots for mixed-effects model assumptions; Figure S2: Prominent gut-associated lymphoid tissue in the cecum; Figure S3: Mild periportal mononuclear infiltrate in the liver; Figure S4: Mild focal mononuclear infiltrate in the kidney; Figure S5: Mild focal mononuclear infiltrate in the pancreas; Figure S6: Representative gross pathological findings in a single bird from the 1× group.

## References

[1] Ajulo S., Awosile B. (2024). Global Antimicrobial Resistance and Use Surveillance System (GLASS 2022): Investigating the Relationship between Antimicrobial Resistance and Antimicrobial Consumption Data across the Participating Countries. PLoS ONE.

[2] No Time to Wait: Securing the Future from Drug-Resistant Infections. https://www.who.int/publications/i/item/no-time-to-wait-securing-the-future-from-drug-resistant-infections.

[3] O’Neill J. (2016). Tackling Drug-Resistant Infections Globally: Final Report and Recommendations.

[4] Mulchandani R., Wang Y., Gilbert M., Van Boeckel T.P. (2023). Global Trends in Antimicrobial Use in Food-Producing Animals: 2020 to 2030. PLoS Glob. Public Health.

[5] (2006). Commission Regulation (EC) No 1177/2006 of 1 August 2006 Implementing Regulation (EC) No 2160/2003 of the European Parliament and of the Council as Regards Requirements for the Use of Specific Control Methods in the Framework of the National Programmes for the Control of Salmonella in Poultry (Text with EEA Relevance). https://eur-lex.europa.eu/eli/reg/2006/1177/oj/eng.

[6] European Food Safety Authority (EFSA), European Centre for Disease Prevention and Control (ECDC) (2023). The European Union One Health 2022 Zoonoses Report. EFSA J..

[7] Andino A., Hanning I. (2015). *Salmonella* Enterica: Survival, Colonization, and Virulence Differences among Serovars. Sci. World J..

[8] Antunes P., Mourão J., Campos J., Peixe L. (2016). Salmonellosis: The Role of Poultry Meat. Clin. Microbiol. Infect..

[9] Jajere S.M. (2019). A Review of Salmonella Enterica with Particular Focus on the Pathogenicity and Virulence Factors, Host Specificity and Antimicrobial Resistance Including Multidrug Resistance. Vet. World.

[10] European Food Safety Authority (EFSA), European Centre for Disease Prevention and Control (ECDC) (2023). The European Union Summary Report on Antimicrobial Resistance in Zoonotic and Indicator Bacteria from Humans, Animals and Food in 2020/2021. EFSA J..

[11] Csitári G., Pál L., Farkas V., Such N., Dublecz K. (2023). Relationship between the egg bacteriota and the chicken gut bacteriota in the light of new-generation sequencing (NGS) methods. Magy. Állatorvosok Lapja.

[12] Kerek Á., Szabó Á., Bányai K., Kaszab E., Bali K., Papp M., Kovács L., Jerzsele Á. (2024). Resistome Analysis of *Escherichia coli* Isolates from Layers in Hungary. Acta Vet. Hung..

[13] Mihailovskaya V.S., Erjavec M.S., Kuznetsova M.V. (2024). *Escherichia coli* from Healthy Farm Animals: Antimicrobial Resistance, Resistance Genes and Mobile Genetic Elements. Acta Vet. Hung..

[14] Máté M., Gombos L., Holes V.F., Ózsvári L. (2026). Changes in antibiotic use in Hungarian large pig farms between 2022 and 2024. Magy. Állatorvosok Lapja.

[15] Sarıçam İnce S., Akan M. (2024). Distribution of antimicrobial resistance and virulence markers in chicken-originated *Proteus mirabilis* isolates. Acta Vet. Hung..

[16] Tanner J.R., Kingsley R.A. (2018). Evolution of *Salmonella* within Hosts. Trends Microbiol..

[17] Hetényi N., Bersényi A., Hullár I. (2024). Physiological Effects of Medium-Chain Fatty Acids and Triglycerides, and Their Potential Use in Poultry and Swine Nutrition: A Literature Review. Magy. Állatorvosok Lapja.

[18] Foley S.L., Johnson T.J., Ricke S.C., Nayak R., Danzeisen J. (2013). *Salmonella* Pathogenicity and Host Adaptation in Chicken-Associated Serovars. Microbiol. Mol. Biol. Rev..

[19] Wernicki A., Nowaczek A., Urban-Chmiel R. (2017). Bacteriophage Therapy to Combat Bacterial Infections in Poultry. Virol. J..

[20] Bardina C., Spricigo D.A., Cortés P., Llagostera M. (2012). Significance of the Bacteriophage Treatment Schedule in Reducing *Salmonella* Colonization of Poultry. Appl. Environ. Microbiol..

[21] Clavijo V., Baquero D., Hernandez S., Farfan J.C., Arias J., Arévalo A., Donado-Godoy P., Vives-Flores M. (2019). Phage Cocktail SalmoFREE^®^ Reduces *Salmonella* on a Commercial Broiler Farm. Poult. Sci..

[22] Żbikowska K., Michalczuk M., Dolka B. (2020). The Use of Bacteriophages in the Poultry Industry. Animals.

[23] Sillankorva S., Pleteneva E., Shaburova O., Santos S., Carvalho C., Azeredo J., Krylov V. (2010). *Salmonella* Enteritidis Bacteriophage Candidates for Phage Therapy of Poultry. J. Appl. Microbiol..

[24] Wójcik E.A., Stańczyk M., Wojtasik A., Kowalska J.D., Nowakowska M., Łukasiak M., Bartnicka M., Kazimierczak J., Dastych J. (2020). Comprehensive Evaluation of the Safety and Efficacy of BAFASAL^®^ Bacteriophage Preparation for the Reduction of *Salmonella* in the Food Chain. Viruses.

[25] Kang H.-W., Kim J.-W., Jung T.-S., Woo G.-J. (2013). Wksl3, a New Biocontrol Agent for *Salmonella enterica serovars Enteritidis* and *Typhimurium* in Foods: Characterization, Application, Sequence Analysis, and Oral Acute Toxicity Study. Appl. Environ. Microbiol..

[26] Kim J.H., Kim H.J., Jung S.J., Mizan M.F.R., Park S.H., Ha S.-D. (2020). Characterization of *Salmonella* spp.-Specific Bacteriophages and Their Biocontrol Application in Chicken Breast Meat. J. Food Sci..

[27] Sevilla-Navarro S., Catalá-Gregori P., García C., Cortés V., Marin C. (2020). *Salmonella* Infantis and *Salmonella* Enteritidis Specific Bacteriophages Isolated Form Poultry Faeces as a Complementary Tool for Cleaning and Disinfection against *Salmonella*. Comp. Immunol. Microbiol. Infect. Dis..

[28] Bampidis V., Azimonti G., Bastos M.d.L., Christensen H., Dusemund B., Kouba M., Durjava M.F., López-Alonso M., López Puente S., EFSA Panel on Additives and Products or Substances used in Animal Feed (FEEDAP) (2021). Safety and Efficacy of a Feed Additive Consisting on the Bacteriophages PCM F/00069, PCM F/00070, PCM F/00071 and PCM F/00097 Infecting *Salmonella* Gallinarum B/00111 (Bafasal^®^) for All Avian Species (Proteon Pharmaceuticals S.A.). EFSA J..

[29] Uddin M.J., Ahn J. (2020). Associations between Antibiotic Resistance and Bacteriophage Resistance Phenotypes in Laboratory and Clinical Strains of *Salmonella enterica* subsp. *enterica* serovar Typhimurium. Microb. Pathog..

[30] Hyman P., Abedon S.T. (2010). Bacteriophage Host Range and Bacterial Resistance. Adv. Appl. Microbiol..

[31] Loc-Carrillo C., Abedon S.T. (2011). Pros and Cons of Phage Therapy. Bacteriophage.

[32] Sulakvelidze A., Alavidze Z., Morris J.G. (2001). Bacteriophage Therapy. Antimicrob. Agents Chemother..

[33] Kutter E., De Vos D., Gvasalia G., Alavidze Z., Gogokhia L., Kuhl S., Abedon S.T. (2010). Phage Therapy in Clinical Practice: Treatment of Human Infections. Curr. Pharm. Biotechnol..

[34] Van Belleghem J.D., Dąbrowska K., Vaneechoutte M., Barr J.J., Bollyky P.L. (2018). Interactions between Bacteriophage, Bacteria, and the Mammalian Immune System. Viruses.

[35] Gindin M., Febvre H.P., Rao S., Wallace T.C., Weir T.L. (2019). Bacteriophage for Gastrointestinal Health (PHAGE) Study: Evaluating the Safety and Tolerability of Supplemental Bacteriophage Consumption. J. Am. Coll. Nutr..

[36] Soffer N., Abuladze T., Woolston J., Li M., Hanna L.F., Heyse S., Charbonneau D., Sulakvelidze A. (2016). Bacteriophages Safely Reduce *Salmonella* Contamination in Pet Food and Raw Pet Food Ingredients. Bacteriophage.

[37] Li M., Lin H., Jing Y., Wang J. (2020). Broad-Host-Range *Salmonella* Bacteriophage STP4-a and Its Potential Application Evaluation in Poultry Industry. Poult. Sci..

[38] Sarker S.A., Sultana S., Reuteler G., Moine D., Descombes P., Charton F., Bourdin G., McCallin S., Ngom-Bru C., Neville T. (2016). Oral Phage Therapy of Acute Bacterial Diarrhea With Two Coliphage Preparations: A Randomized Trial in Children From Bangladesh. EBioMedicine.

[39] Brüssow H. (2012). What Is Needed for Phage Therapy to Become a Reality in Western Medicine?. Virology.

[40] Chan B.K., Abedon S.T., Loc-Carrillo C. (2013). Phage Cocktails and the Future of Phage Therapy. Future Microbiol..

[41] Abedon S.T., Kuhl S.J., Blasdel B.G., Kutter E.M. (2011). Phage Treatment of Human Infections. Bacteriophage.

[42] Percie du Sert N., Hurst V., Ahluwalia A., Alam S., Avey M.T., Baker M., Browne W.J., Clark A., Cuthill I.C., Dirnagl U. (2020). The ARRIVE Guidelines 2.0: Updated Guidelines for Reporting Animal Research. PLoS Biol..

[43] R Core Team (2020). R: A Language and Environment for Statistical Computing.

[44] Bates D., Mächler M., Bolker B., Walker S. (2015). Fitting Linear Mixed-Effects Models Using Lme4. J. Stat. Softw..

[45] Hothorn T., Bretz F., Westfall P. (2008). Simultaneous Inference in General Parametric Models. Biom. J..

[46] Dunnett C.W. (1955). A Multiple Comparison Procedure for Comparing Several Treatments with a Control. J. Am. Stat. Assoc..

[47] Pinheiro J., Bates D. (2000). Mixed-Effects Models in S and S-PLUS.

[48] Pintér K., Pollák B.D., Gantelet H., Magyar T. (2024). Antimicrobial Susceptibility of *Pasteurella multocida* Strains Isolated from Different Host Species. Acta Vet. Hung..

