# Supplementary material for: Formulation-Level Tolerability of Repeated Drinking-Water Administration of a Commercial Multi-Component Bacteriophage Product in Ross 308 Broiler Chickens: A Pilot 27-Day Dose-Escalation Study
Source: Vet Sci. 2026 Jul 24;13(8):730. doi: 10.3390/vetsci13080730 (PMC13517655; doi:10.3390/vetsci13080730)
Supplement: Supplementary file 1 [file vetsci-13-00730-s001.zip › Supplementary materials.pdf]

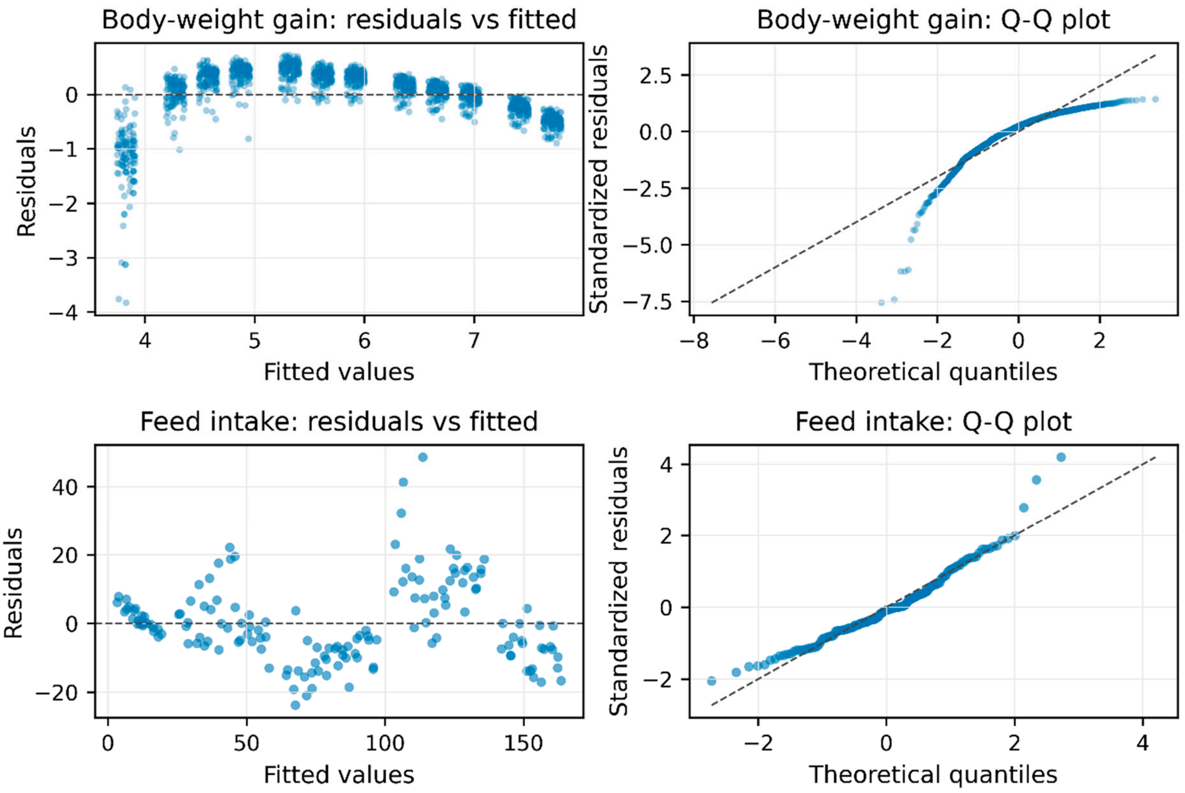

Figure S1. Diagnostic plots for mixed-effects model assumptions. Diagnostic plots used to assess the assumptions of the linear mixed-effects models fitted to body-weight gain and pen-level feed intake. The plots include residual-versus-fitted-value displays and normal quantile–quantile plots for the model residuals. Body-weight gain was analyzed after logarithmic transformation, whereas feed intake was analyzed on the original g/bird/day scale. The diagnostic plots did not indicate major violations that would alter the interpretation of the treatment comparisons.

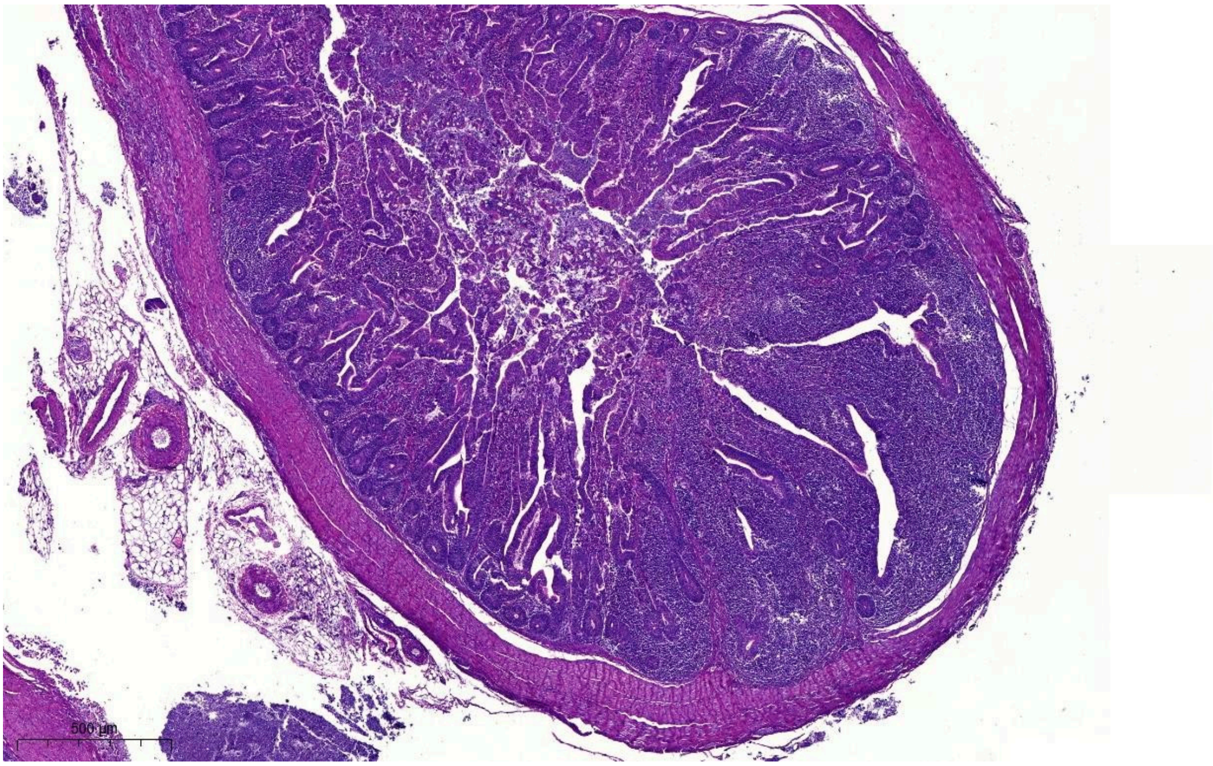

Figure S2. Prominent gut-associated lymphoid tissue in the cecum. Representative H&E-stained cecal section.

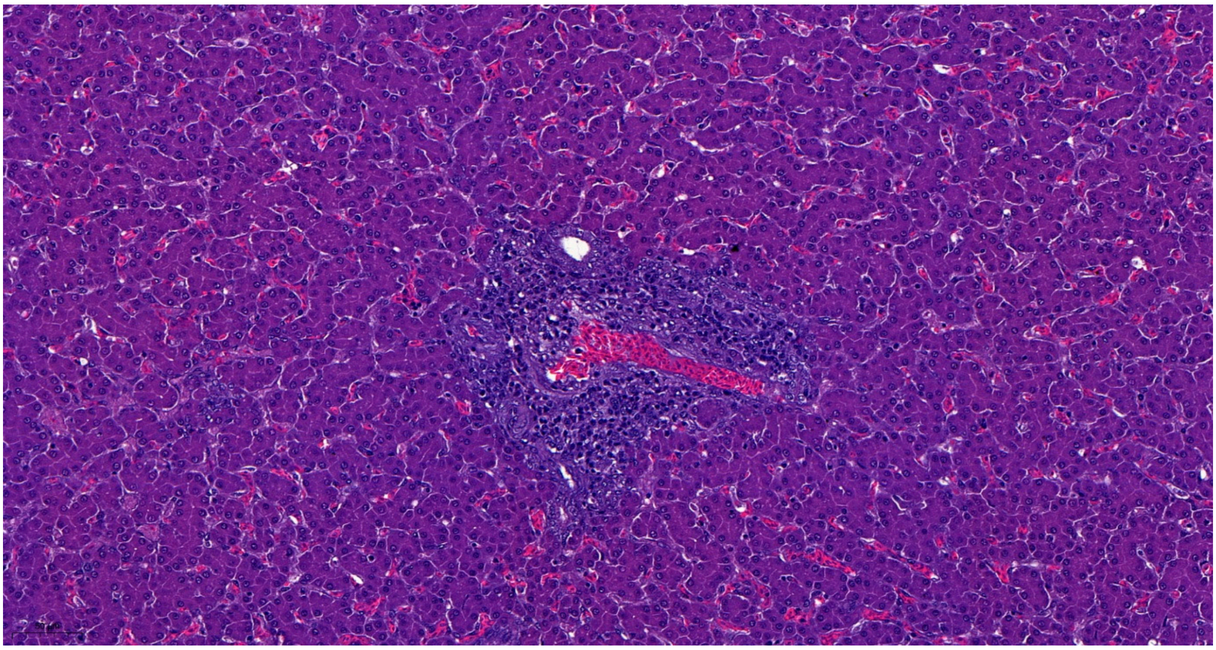

Figure S3. Mild periportal mononuclear infiltrate in the liver. Representative H&E-stained liver section.

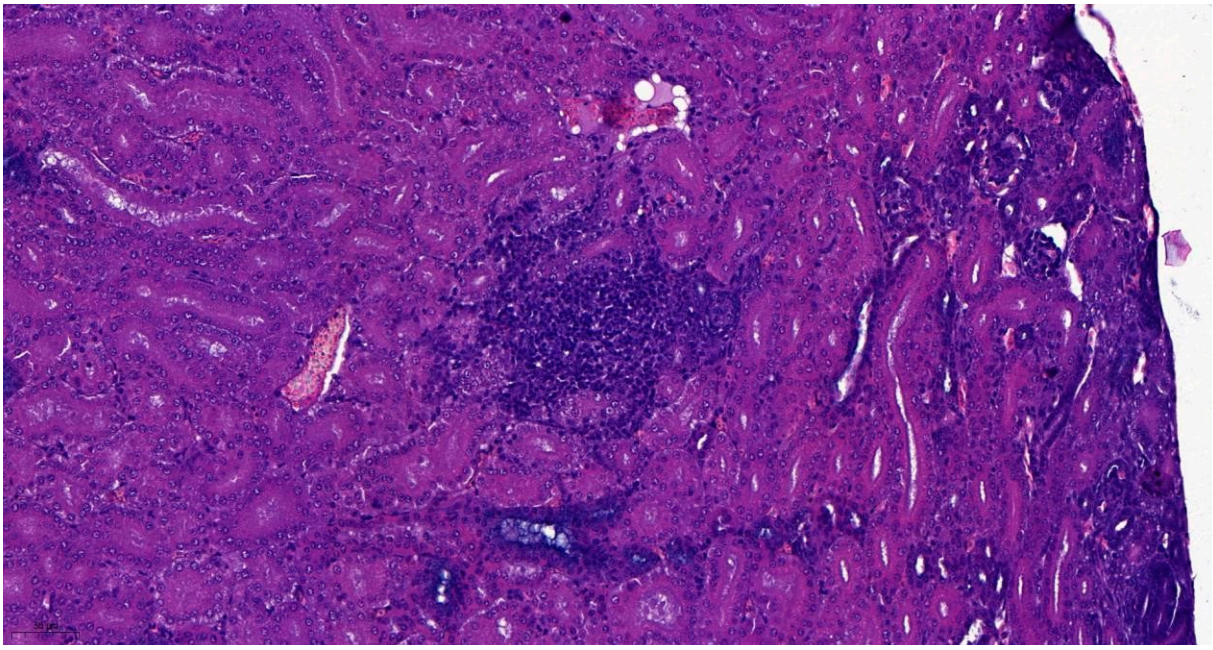

Figure S4. Mild focal mononuclear infiltrate in the kidney. Representative H&E-stained kidney section.

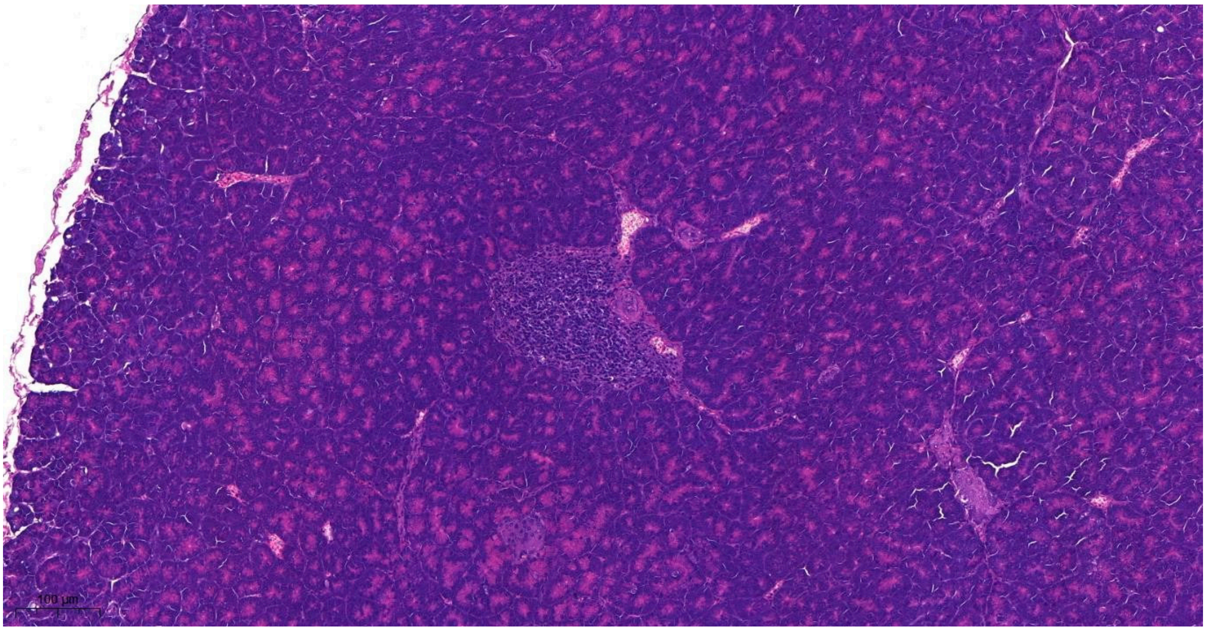

Figure S5. Mild focal mononuclear infiltrate in the pancreas. Representative H&E-stained pancreatic section.

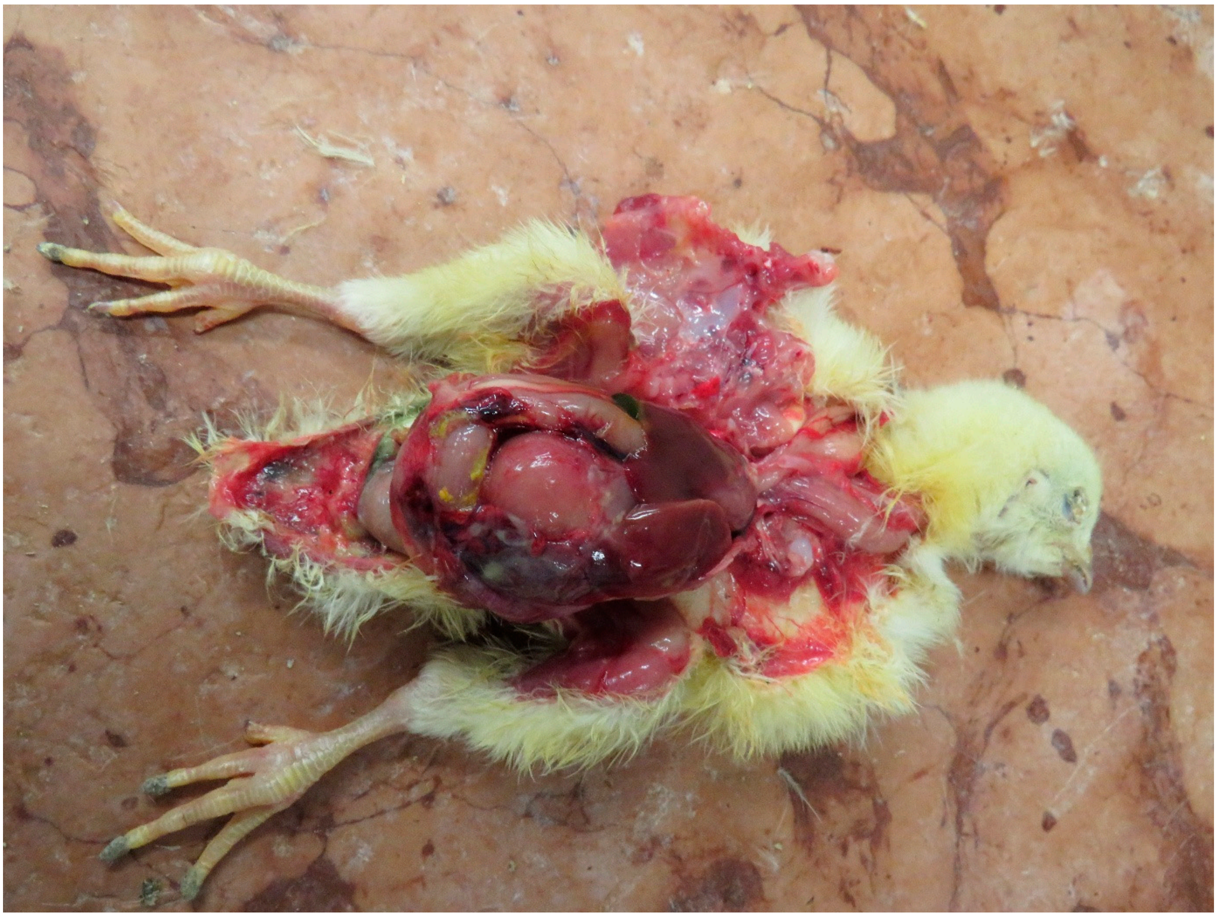

Figure S6. Representative gross pathological findings in a single bird from the 1× group. Gross postmortem image showing marked inflammatory lesions involving visceral organs and serosal surfaces. In the context of the overall study, this case was considered an incidental individual finding and was not interpreted as evidence of a treatment-related or dose-dependent pathological pattern.
